# Diagnostic Value of Muscle MRI in a Case of Very Late-Onset Becker Muscular Dystrophy

**DOI:** 10.1007/s00062-026-01620-0

**Published:** 2026-02-05

**Authors:** Evamaria O. Riedel, Vincent Gmeiner, Tobias B. Haack, Jan S. Kirschke, Marcus Deschauer

**Affiliations:** 1 Department Neuroradiology, School of Medicine and Health, TUM Klinikum Rechts der Isar, Technical University of Munich, Munich, Germany; 2 Department of Neurology, School of Medicine and Health, TUM Klinikum Rechts der Isar, Technical University of Munich, Munich, Germany; 3 Institute for Medical Genetics and Applied Genomics, Eberhard Karls University, Tübingen, Germany; 4 Center for Rare Diseases, Eberhard Karls University, Tübingen, Germany

## Introduction

The diagnostic evaluation of myopathies can be challenging, particularly in adults and elderly patients with comorbidities and atypical clinical presentations. While a few hereditary myopathies such as myotonic dystrophy types 1 (DM1) and 2 (DM2) or facioscapulohumeral muscular dystrophy (FSHD) present with sufficiently characteristic phenotypes to allow targeted genetic testing, most patients exhibit limb-girdle weakness with a broad differential diagnosis. In older individuals, acquired conditions—especially idiopathic inflammatory myopathies—are often considered first. But also late-onset hereditary myopathies, including autosomal-dominant and autosomal-recessive limb-girdle muscular dystrophies (LGMDs) as well as the x‑linked rarely late-manifesting Becker muscular dystrophy, which typically presents in adolescence or early adulthood, must be considered.

When clinical classification remains uncertain, muscle MRI is increasingly used to distinguish inflammatory from hereditary myopathies. However, assigning a specific hereditary myopathy based on MRI remains often difficult, even though characteristic patterns of muscle involvement have been described for some of them [1, 2]. Regarding dystrophinopathies, muscle MRI is well established in boys with Duchenne muscular dystrophy (DMD), but considerably fewer data exist on MRI findings in Becker muscular dystrophy (BMD). In particular, MRI characteristics in elderly patients with atypical presentations remain underreported.

We describe a patient with symptom onset at 64 years of age—an age at which dystrophinopathies are seldom considered—whose diagnosis was guided by muscle MRI demonstrating a selective pattern of fatty infiltration characteristic of Becker dystrophinopathy. The diagnosis was subsequently confirmed by a rare hemizygous pathogenic variant in the dystrophin gene (*dmd*; c.9G > A, p.Trp3Ter), consistent with a mild, very late-onset BMD phenotype.

## Case Presentation

A 70-year-old male presented with a six-year history of slowly progressive, symmetric muscle weakness in the lower limbs, retrospectively first noted at age 64 when ascending stairs or ladders during physically demanding work as an installation technician. Symptoms had progressed slowly, prompting medical evaluation at age 69. Upon presentation in our clinic at age 70, his walking distance was limited to approximately 1000 m and stair climbing to one flight. Over the preceding six months, he had also noted progressive weakness in arm flexion. Mild exertional dyspnea was reported.

Medical history included a smoking history of 70 pack-years, pre-existing arterial hypertension, and coronary artery disease. There was no family history of muscle weakness among both parents, two brothers, one son and one daughter.

Neurological examination revealed slight atrophy of the legs, symmetric tetraparesis predominantly affecting the lower limbs, waddling gait, difficulty rising from a seated position and a positive Gower’s sign (***Suppl. Video 1***). Manual muscle testing showed bilaterally reduced strength (Medical Research Council (MRC) scale 4/5) in upper arm abduction, elbow flexion, hip extension, hip flexion, and knee flexion. Cardiac evaluation, including transthoracic echocardiography and electrocardiogram (ECG) was unremarkable. Vital capacity was normal (4.3 L). Diabetes or cataract were absent.

Laboratory testing revealed persistently mild elevated creatine kinase (CK, 400–500 U/L) despite discontinuation of statin therapy. Needle Electromyography (EMG) demonstrated positive sharp waves, fibrillations, and recurrent myotonic discharges in the left medial gastrocnemius with normal motor unit potentials.

**Fig. 1:**
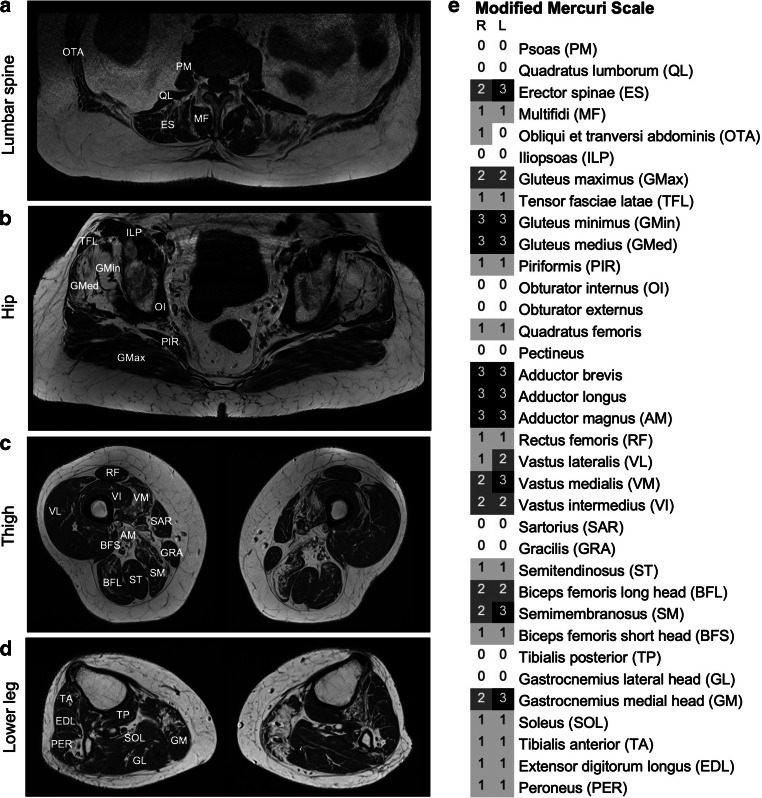
*T1-weighted muscle MRI of the lower extremities.* Axial T1‑w TSE images at the level of the lumbar spine (**a**), hip (**b**), thigh (**c**), and lower leg (**d**) demonstrate fatty atrophy most pronounced in the erector spinae, glutei, adductors, vasti, biceps femoris long head, semimembranosus, and medial gastrocnemius. **e** Modified Mercuri scores for individual muscles (*R* = right, *L* = left), are visualized as a heatmap

The combination of slowly progressive symmetric weakness, mild hyperCKemia, nonspecific EMG findings, and diagnostic uncertainty between inflammatory and hereditary myopathy prompted muscle MRI which played a pivotal role in diagnosis. Imaging of the lower extremities on a Siemens 1.5 T scanner revealed a characteristic dystrophic pattern of fatty infiltration, most pronounced with modified Mercuri score (MMS) 2–3 in the erector spinae, glutei, adductors, vasti, biceps femoris long head, semimembranosus, and medial gastrocnemius (Fig. 1). Muscles showing milder involvement (MMS 1) were the multifidi, obliqui et transversi abdominis, tensor fasciae latae, piriformis, quadratus femoris, rectus femoris, semitendinosus, biceps femoris short head, soleus, tibialis anterior, extensor digitorum longus, and peroneus. Maximum side-to-side difference was one point. Minimal edema was observed in the right vastus medialis, left semimembranosus, and right medial gastrocnemius, which supported a chronic rather than inflammatory process.

Genome sequencing identified a hemizygous pathogenic variant in exon 1 of *DMD* (c.9G > A, p.Trp3Ter).

## Discussion

The combination of progressive, symmetric proximal muscle weakness, spontaneous activity on EMG, mild hyperCKemia, absence of a family history of muscle disease, and advanced age initially suggested an immune-mediated inflammatory myopathy, such as inclusion body myositis (IBM). However, muscle MRI findings were not consistent with IBM, which typically shows a “melted” appearance of the vastus intermedius and medialis muscles, and predominant involvement of the anterior compartment compared to the posterior in the distal thigh [3].

Myotonic discharges on EMG and clinical features also raised suspicion for DM2. However, myotonic discharges can be observed not only in myotonia (dystrophic or non-dystrophic) but also in various other hereditary myopathies, occasionally in BMD [4]. MRI argued against DM2, for which no characteristic MRI pattern is established; mostly normal findings, or occasional fatty degeneration primarily present in the erector spinae, followed by the gluteus maximus, with consistent sparing of rectus femoris and gracilis are described [5]. Although this pattern does not exclude DM2, the MRI findings in our patient—with selective fatty degeneration and minimal edema—was decisive in differentiating a muscular dystrophy from inflammatory or myotonic disorders.

Muscle MRI in BMD typically reveals a selective pattern of muscle involvement resembling that seen in DMD, but less severe. The gluteus maximus and medius, adductor magnus, vasti, biceps femoris long head, and semimembranosus are usually most affected [6, 7], and our patient showed MMS 2–3 in all these muscles. The soleus, peroneus, and obliqui et transversi abdominis are described as frequently but mildly involved, consistent with the mild involvement in our patient (MMS 1). Iliopsoas, obturator externus, sartorius, gracilis, tibialis posterior, tibialis anterior, and the anterolateral leg compartment are generally relatively spared; in our patient, most of these muscles were spared (MMS 0), while tibialis anterior and the anterolateral compartment showed mild involvement (MMS 1). Additional characteristic features, which were also present in our patient, include greater lateral than medial involvement of paraspinal muscles and generally symmetric muscle involvement, with a maximum side-to-side difference of one point [6]. To our knowledge, no MRI studies have previously been reported in patients carrying the p.Trp3Ter variant [8].

Given the suspicion of a hereditary myopathy with very late-onset, supported by MRI findings, genome sequencing was performed. This revealed a pathogenic variant in *DMD* (ENST00000357033.9: c.9G > A, p.Trp3Ter). This truncating mutation in exon 1 would be expected to result in severe DMD due to an missing protein containing just two amino acids. However, the clinical phenotype is associated with a very mild phenotype of BMD [8]. The molecular mechanism responsible for the amelioration of disease severity is initiation of translation at two alternative start codons within exon 6. This results in a shorted protein with missing translation of exon 1–5. It was concluded that domains encoded within the first five exons of dystrophin are not necessary in order to maintain some residual function of the protein [9]. To our knowledge, this variant has been described in seven BMD families with a rather mild phenotype of BMD starting frequently in childhood. Some family members carrying the variant were oligosymptomatic in late adulthood [8]. Due to this “mild” *DMD* variant presentation of BMD in our patient was notably later, at 69 years of age, with retrospective symptom onset at 64 years. Likewise, a larger BMD cohort [10] reports symptom onset between 0.8 and 51 years, highlighting the rarity of such very late-onset presentations.

Cardiomyopathy is observed in 17–74% of patients with BMD [10, 11], but it was not present in our patient nor in the other reported cases carrying the p.Trp3Ter variant [8].

Currently, therapy of BMD remains purely symptomatic with particular emphasis on drug treatment for cardiomyopathy. In contrast to Duchenne muscular dystrophy, steroid therapy is generally not recommended in BMD. No disease-modifying or gene-specific therapy is available at present [10, 12].

## Conclusion

This case illustrates the pivotal role of muscle MRI as a non-invasive diagnostic tool in diagnosing hereditary myopathies in an elderly patient with unexplained muscle weakness. In patients whose clinical presentation does not suggest a clear phenotype attributable to a specific hereditary myopathy, deeper phenotyping through muscle MRI can be particularly valuable. Even in older individuals with an unremarkable family history—where a genetic muscle disease may not be suspected—MRI can reveal characteristic patterns of involvement and guide further diagnostic workup prompting early genetic testing and preventing a muscle biopsy. Our case highlights the diagnostic value of muscle MRI in such a rare, very late-onset case of BMD and underscores the diagnostic utility of muscle MRI in atypical dystrophinopathies.

## Supplementary Information

ESM1: Supplementary material 1
